# Mechanobiological Approach for Intestinal Mucosal Immunology

**DOI:** 10.3390/biology14020110

**Published:** 2025-01-22

**Authors:** Hyeyun Kim, Se-Hui Lee, Jin-Young Yang

**Affiliations:** 1 Department of Integrated Biological Science, Pusan National University, Busan 46241, Republic of Korea; kelly5060@pusan.ac.kr (H.K.); two_hui@pusan.ac.kr (S.-H.L.); 2 Institute for Future Earth, Pusan National University, Busan 46241, Republic of Korea; 3 Department of Biological Sciences, Pusan National University, Busan 46241, Republic of Korea

**Keywords:** intestinal stem cells, shear stress, compression, stretch, stiffness, mechanical stress, cell proliferation

## Abstract

The intestinal area is composed of diverse cell types that harmonize gut homeostasis, which is influenced by both endogenous and exogenous factors. Notably, the environment of the intestine is exposed to several types of mechanical forces, including shear stress generated by fluid flow, compression and stretch generated by luminal contents and peristaltic waves of the intestine, and stiffness attributed to the extracellular matrix. These forces play critical roles in the regulation of cell proliferation, differentiation, and migration. Many efforts have been made to simulate the actual intestinal environment in vitro. The three-dimensional organoid culture system has emerged as a powerful tool for studying the mechanism of the intestinal epithelial barrier, mimicking rapidly renewing epithelium from intestinal stem cells (ISCs) in vivo. However, many aspects of how mechanical forces, such as shear stress, stiffness, compression, and stretch forces, influence the intestinal area remain unresolved. Here, we review the recent studies elucidating the impact of mechanical forces on intestinal immunity, interaction with the gut microbiome, and intestinal diseases.

## Simple Summary

The intestinal epithelium is a highly dynamic space composed of several cell types, each with distinct functions that contribute to gut maintenance and homeostasis. Intestinal epithelial cells include enterocytes for nutrient absorption, Paneth cells that secrete antimicrobial peptides, goblet cells for mucus secretion, enteroendocrine cells for hormone release, and intestinal stem cells for proliferation. They interact with each other to maintain intestinal epithelial barrier function. The extracellular matrix and several mechanical forces such as shear stress, compression, and stretching influence their function, and the dysregulation of their function contributes to intestinal diseases. Understanding the interplay between intestinal epithelial cells and mechanical forces could help in the development of new therapeutic strategies for intestinal diseases.

## 1. Intestinal Microenvironment

### 1.1. Importance of the Intestine

The intestine is a vital organ responsible for digestion, nutrient absorption, and protection against pathogens. Its surface is lined with intestinal epithelial cells (IECs), which are composed of diverse cell types that cluster together to form a physical barrier and numerous finger-like villi [1]. Longer finger-like villi, which significantly expand the surface area to improve nutrient absorption and increase the opportunity for interaction with microorganisms, are prominent in the small intestine [2]. The small intestine is responsible for breaking down food into carbohydrates, proteins, and fats. Some diseases or disorders of the small intestine, such as celiac disease, Crohn’s disease, irritable bowel syndrome (IBS), and enteritis, disrupt its function [3,4]. The large intestine, also known as the colon, exhibits shorter villi but well-organized crypts [2]. Its primary function is to absorb water, electrolytes, and vitamins, such as K and B. The large intestine also excretes waste from the feces [5]. These functions are usually compromised by inflammatory diseases of the colon, such as colitis [6]. The structural differences between the small and large intestines allow each segment of the gut to perform its functions effectively.

The intestine is not only a site for nutrient absorption but also a critical organ for the immune system with organized lymphoid tissues and a large population of immune cells [7]. The most well-known lymphoid structure in the intestine is gut-associated lymphoid tissue (GALT). It contains Peyer’s patches (PPs) and isolated lymphoid follicles (ILFs), serving as a key site for antigen sampling and immune cell activation. PPs, predominantly located in the ileum, and ILFs contain microfold (M) cells, delivering antigens to antigen-presenting cells (APCs) including dendritic cells and macrophages, which process the antigens and present them to naïve T and B cells [8,9]. Also, PPs and ILFs have B-cell follicles containing germinal centers (GCs). Within GCs, B cells undergo class-switch recombination and somatic hypermutation, and when an antigen is presented to B cells, it enhances immunoglobulin (Ig) A production [10,11]. IgA is the major immunoglobulin isotype in the intestine, and secreted IgA acts as the first line of defense protecting mucosal surfaces from microbial invasion. It neutralizes bacterial toxins and viruses; blocks bacterial adherence, translocation, and invasion of epithelial cells; and regulates gut microbiota function [12,13]. In addition to the GALT, IECs contribute to the immune system through pattern recognition receptors (PRRs) including Toll-like receptors (TLRs), and NOD-like receptors (NLRs) [14]. PRRs recognize the pathogen-associated molecular patterns (PAMPs) of microbial agents including lipopolysaccharides (LPS) and initiate intracellular signaling pathways [15,16]. Through these mechanisms, the gut can regulate immune responses, maintain tolerance to commensal microbes and antigens, and maintain homeostasis. The harmonization of GALT with APCs, IgA production, and signaling pathways regulated by PRRs allows the gut to act as a barrier to pathogens and immune regulators, preserving the integrity of the mucosal environment.

In addition to their contribution to the immune system, the intestine is a major habitat for the gut microbiome, which comprises more than 100 trillion bacteria, viruses, and fungi. They help maintain the intestinal epithelial barrier and have anti-inflammatory functions [17]. The gut microbiome is influenced by many factors including diet, age, geography, radiotherapy, and medicine. It is well known that dysbiosis of the gut microbiome is associated with intestinal diseases, and intestinal diseases can be ameliorated by treatment with probiotics or fecal microbiome transplantation, suggesting the important role of the gut microbiome [18,19]. In addition, the microbiome regulates metabolic processes and systemic health including diabetes, obesity, and digestion [20,21]. Beyond the function themselves, the secreted metabolites such as short-chain fatty acids (SCFAs) like butyrate, acetate, and propionate and extracellular vesicles also regulate gut homeostasis and circulate in the host body, affecting other organs. For example, it has been revealed that SCFAs impact the sympathetic nervous system, hormone release, and memory processes, indicating the importance of the gut–brain axis [22]. Furthermore, SCFAs influence the inflammation of the skin and lungs showing a gut–skin and gut–lung axis [23,24,25]. This gut–organ axis underscores the importance of the microbiome.

The intestine is known for its rapid regenerative ability. Stem cells located at the base of crypts regenerate new epithelial cells every three to five days. Owing to this rapid turnover capacity, damaged or aged cells can be continuously replaced, preserving the function and integrity of the epithelial barrier [26]. The process is tightly regulated by the Wnt/β-catenin signaling pathway and expression level of bone morphogenic protein (BMP). For example, Wnt activation is related to the maintenance of stem cell populations, whereas BMP promotes the differentiation of stem cells migrating to the villi [27]. Signaling pathways such as the Notch and Yes-associated protein (YAP)/transcriptional co-activator with PDZ-binding motif (TAZ) pathways also regulate the balance between cell proliferation and differentiation [28]. Disruptions in this regenerative process can lead to abnormal stem cell proliferation or differentiation, resulting in colorectal cancer [29]. Moreover, the intestine is constantly exposed to various mechanical forces generated by the movement of intestinal contents or extracellular matrix (ECM) remodeling. These mechanical forces influence epithelial cell behavior, such as proliferation, differentiation, and migration, and play a critical role in maintaining intestinal homeostasis [30]. The altered mechanical force changes the normal cell signaling pathways, such as the Wnt/β-catenin signaling pathway, and the YAP/TAZ signaling pathway, contributing to the disruption of intestinal homeostasis [28,30].

In summary, the intestine exhibits a structural complexity and regenerative capacity, acts as a physical barrier, provides a home for the gut microbiome, and regulates mechanical forces. These characteristics synergistically maintain the overall health and function of the intestine. Understanding the mechanisms underlying intestinal integrity and function is essential for regulating gastrointestinal disorders and improving systemic well-being.

### 1.2. Components of Intestinal Barrier

The large and small intestines act as selective barriers, and they regulate nutrient flow while preventing harmful pathogens, toxins, and antigens [31]. The function of this barrier is maintained by tight junctions, adherent junctions, and desmosomes between epithelial cells, which play crucial roles in intestinal integrity [32]. The intestinal barrier is composed of a variety of epithelial cells (Table 1), including goblet, tuft, enteroendocrine, Paneth, and intestinal stem cells (ISCs). These cells are interconnected by tight junctions, which maintain homeostasis by protecting them against microbial invasion and acting as a physical barrier [33] 

Goblet cells are present at particularly high frequencies in the large intestine, where they secrete mucin and form a mucus layer. The mucus layer protects the intestinal epithelium from mechanical damage and acts as a barrier against pathogens [34]. Tuft cells, a rare steady-state cell type, are chemosensory cells that participate in the Th2 response. They proliferate when infected by parasites and release immune factors, such as cytokines and chemokines [35]. In particular, they produce interleukin (IL)-25, which further drives tuft cell expansion and response to parasites [36,37]. Paneth cells are present at the base of the small intestinal crypts and secrete antimicrobial peptides (AMPs), such as defensins. These peptides play a role in the defense against bacteria and other microbes in the intestinal lumen. Enteroendocrine cells produce and release a range of gut hormones that play key roles in digestion and absorption [38]. Food digestion or metabolites in the gut microbiome stimulate the production of cholecystokinin (CCK) and secretin [39]. Microfolds or M cells are specialized epithelial cells that transport antigens from the lumen to immune cells in the Peyer’s patches, contributing to the immune surveillance of the gut [40]. ISCs are located in the intestinal crypts and continually divide and differentiate into various cell types, renewing the intestinal epithelium. ISCs are responsible for the continuous renewal of the intestinal epithelial barrier and the replacement of damaged or lost cells in the intestinal epithelium. They divide and move to the transit-amplifying zone (TA) to reach the differentiation zone [41]. This process helps maintain the integrity of the intestinal epithelial barrier and homeostasis and ensures its proper functions, such as the absorption of nutrients, digestion, and protection against pathogens in the host. All IECs function together to develop an intestinal barrier to protect the intestine from pathogens, toxins, and food while also serving as a habitat for the gut microbiome [42]. The disruption of gut homeostasis can lead to IBDs, including ulcerative colitis and Crohn’s disease [43]. Therefore, understanding the differentiation and renewal processes of the intestinal epithelial barrier is essential to protect against intestinal diseases.

**Table 1:** Types and functions of intestinal epithelial cells.

Cell Type	Location	Key Functions	References
**Enterocytes**	Major cell type in intestinal villi	Nutrient absorption Immunomodulator	[44,45]
**Goblet cells**	Scattered in intestinal villi and crypts Prevalent in the large intestine	Mucus secretion Protection against bacterial invasion	[46,47]
**Paneth cells**	Crypts at the base of the small intestine	Production and secretion of AMP Performing phagocytosis, efferocytosis Preserving barrier integrity	[48,49,50]
**Enteroendocrine cells**	Relatively rare throughout the intestine	Release of hormones (GLP-1, GIP, CCK, secretin)	[51]
**M cells**	Follicle-associated epithelium of intestinal Peyer’s patches	Transporting antigen to mucosal lymphoid tissue	[52,53]
**Tuft cells**	Scattered in intestinal villi	Secretion of interleukin-25 after parasitic infection Initiating a type 2 immune response Sensing bacterial metabolite N-undecanoyl glycine	[54,55]
**Stem cells**	Bottom of the intestinal crypt between Paneth cells	Proliferation, and differentiation to replace intestinal epithelium Interacting with gut microbiota	[56,57,58]

## 2. Mechanical Stress in the Intestine

**Figure 1:**
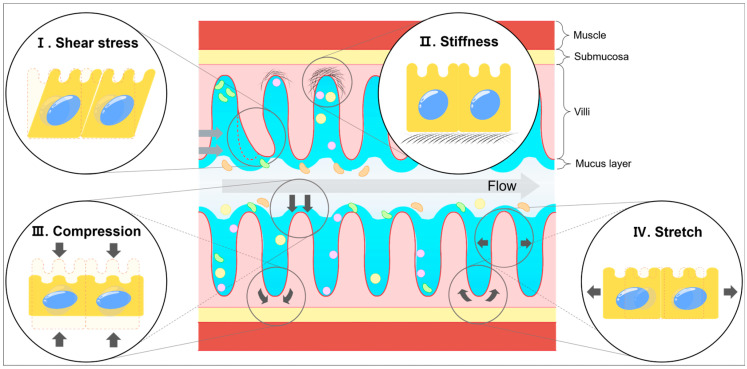
Types of mechanical strains in the intestine. The fluid flow and digestion leads to shear stress (**I**), compression (**III**), and stretch (**IV**). Stiffness (**II**) represents the cross-linking status between ECM components. These mechanical strains influence ISC behavior in various ways. The arrows indicate the direction of the force.

The intestine is a highly dynamic tissue responsible for digestion, protection from pathogens, and many other physical functions. Mechanical forces are generated by fluid flow, peristaltic movements, luminal pressure, and interactions between ECM components [59]. These forces induce mechanical stresses, such as shear stress, stiffness, compression, and stretch stress (Figure 1), and are involved in mechanotransduction pathways that influence cell behavior [60]. Le et al. revealed that multiple physical forces, including compression, osmotic pressure, matrix rigidity, and stretching, can alter the properties of Lgr5^+^ stem cells and regulate the growth of intestinal organoids. Furthermore, these physical forces enhance the Wnt/β-catenin signaling [61]. These mechanical stresses are associated with intestinal diseases; dysregulated mechanical stress compromises the function of IECs, particularly ISCs, leading to intestinal diseases [62]. Therefore, understanding how mechanical stress influences intestinal epithelial cells is crucial for intestinal homeostasis research and provides therapeutic strategies for intestinal disorders.

### 2.1. Shear Stress

Water comprises more than half of the human body. The flow of the fluid leads to shear stress, which affects cellular surfaces. In the intestine, shear stress represents the frictional force between the luminal contents and the intestinal barrier, and it impacts IECs via the flow of digesta [63]. To investigate and control shear stress in the intestine, most studies have utilized the gut-on-chip model under a controlled flow rate. Shear stress plays an important role in the intestine by regulating cell differentiation and maintaining intestinal barrier function [64].

In some studies, the use of a gut-on-chip model to generate fluid flow and expose intestinal cells to shear stress has demonstrated the upregulation of the expression of proteins critical for cell differentiation, epithelial integrity, and intestinal barrier function. According to Yang et al., using Caco-2 cells grown on a gut-on-a-chip under the influence of the fluid flow and cyclic mechanical strain, the catalytic activity of alkaline phosphatase, which is secreted by brush marginal microvilli [65], was greatly increased (Table 2). In addition, the aforementioned proteins form an epithelium alongside well-developed tight junction proteins, such as ZO-1, and brush border proteins, such as ezrin [66]. Another recent study conducted using enteroids cultured in the presence of shear stress showed high expression levels of sucrase-isomaltase and downregulated adenoma, which are well expressed in differentiated enteroids, and low levels of NKCC1, an ion transport protein, which is enriched in undifferentiated enteroids. In addition, the mRNA expression levels of LGR5, a stem cell marker, and Ki67, a proliferation marker, decreased. Based on these results, we can infer that shear stress can accelerate cytodifferentiation and augment the integrity and barrier function [67]. Moreover, a recent study in Drosophila revealed that the transient receptor potential A1 (TrpA1) specifically responds to shear stress and promotes the proliferation and differentiation of ISCs [68]. The midgut of Drosophila is similar to the mammalian gut, and it has been reported that the mammalian gut has high expression levels of TrpA1 behaving similarly to Drosophila TrpA1 [69,70]. These findings suggest that TrpA1 may serve as a mechanosensor in the mammalian gut, offering a promising target for studying the impact of shear stress on ISC behavior.

**Figure 2:**
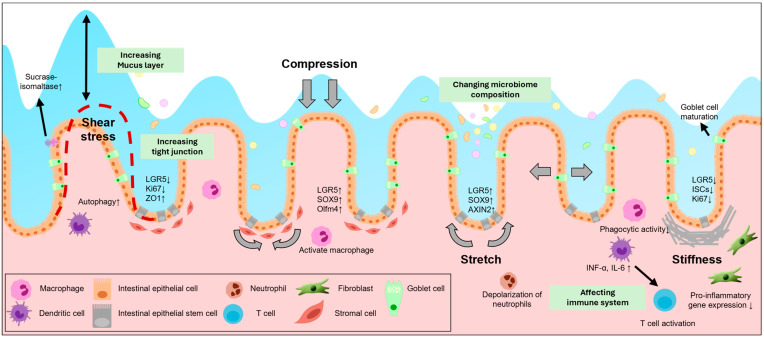
Complex interactions between mechanical forces and various cell types. Mechanical forces affect various cells in the intestine and alter the intestinal environment. These forces are orchestrated in the gut and contribute to intestinal homeostasis in a complex manner, including cell differentiation, proliferation, and immune regulation.

Several studies have suggested that shear stress in the intestine influences cell fate and maintains gut homeostasis. The specific mechanisms are still being examined. However, some studies have shown that shear stress in other organs controls the Notch signaling or Piezo protein, leading to the determination of the fate of stem cells [71,72]. Shear stress also influences the immune system. Studies have demonstrated that shear stress induces autophagy by forming vacuoles in the intestine [63]. Furthermore, caco-2 cells cultured under fluidic conditions have shown increased mucin-2 secretion compared to cells cultured in static conditions [64]. Since mucin contributes to maintaining intestinal barrier function and shear stress activates autophagy and inhibits inflammation in vascular endothelial cells, it seems like shear stress also regulates the immune system in the intestine [73]. Moreover, some researchers using a gut-on-chip model have revealed that increased shear stress can detach the microbiome from Caco-2 cells, while decreased shear stress leads to rapid growth of the microbiome [74]. These findings suggest that shear stress influences microbiome adhesion and growth, showing the potential impact of shear stress on microbiome composition. Through these results, we can infer that shear stress affects not only the behavior of ISCs but also the immune system and microbiome (Figure 2). However, the exact mechanisms and pathways remain unclear. Further studies are needed to elucidate the exact relationship, including the effects of shear stress on immune cell activation, cytokine production, and microbiome composition.

**Table 2:** Several types of mechanical forces and their associated signaling pathways acting on the small and large intestines.

Mechanical Force	Target Organ	Signaling Pathways	Effects on Cells	Reference
Shear stress	Small intestine	Unknown	Unknown	
	Large intestine	Wnt/β-catenin signaling	Form villi-like epithelium	[63,65,66,67,75]
		Autophagy pathway	Form vacuole	
		Unknown	Increase alkaline phosphatase	
			Enhance barrier function	
Stiffness	Small intestine	YAP/TAZ signaling	Goblet cell differentiation	[76,77,78]
		Wnt/β-catenin signaling	Decrease LGR5, Ki67 expression	
			Enhance crypt fission	
	Large intestine	Ca^2+^ dependent	ISCs differentiation	[79,80,81,82]
		Hippo and YAP/TAZ signaling	Uncontrolled proliferation	
		FAK signaling	Enhance fibroblast density Reduce pro-inflammatory genes FAK-dependent proliferation	
Compression	Small intestine	Wnt/β-catenin signaling	Increase LGR5, SOX9 expression	[61]
	Large intestine	Unknown	Unknown	
Stretch	Small intestine	Piezo1-mediated Ca^2+^ influx	Crypt fission	[83,84,85]
		Wnt/β-catenin signaling	Increase Ki67, SOX9 expression	
			Increase LGR5, Olfm4 expression	
		Piezo2-mediated Ca^2+^ influx	Release serotonin	
	Large intestine	Piezo2-mediated Ca^2+^ influx	Release serotonin	[86]

### 2.2. Stiffness

ISCs surround mesenchymal cells, fibroblasts, and myofibroblasts and regulate their proliferation and differentiation. These cells form the ISC niche and a physical niche known as the ECM [87]. The ECM influences cell behavior in various ways, including stiffness. Stiffness is commonly used to represent the resistance of a cell or the deformation of the ECM caused by forces or stresses [88]. Increased tissue stiffening in the intestine is a hallmark of IBD, suggesting that stiffness mediates ISC dysfunction and disrupts homeostasis [76]. Therefore, understanding how the stiffness of the intestinal microenvironment affects the intestine is crucial. However, in vivo experiments determining the effect of ECM stiffness on ISCs are challenging. Therefore, most previous studies have used organoids.

Evidence suggests that stiffness changes cell characteristics such as area, volume, and maturation. In addition, cells, including stem cells, grown on a stiff substrate proliferate more than cells grown on a soft substrate [89,90]. Stiffness also affects the fate of ISCs. A recent study using a 2.5D gut organoid reported that stiffness led to a high expression of YAP, a mechanotransducer, and promoted its translocation into the nucleus. YAP activation negatively correlates with LGR5^+^ ISCs and Ki67^+^ proliferating cells, whereas mature goblet cells positively correlate with YAP activation (Table 2) [76]. Ca^2+^, a secondary messenger, is involved in cell proliferation and differentiation [91]. In another study, organoids grown with stiffer matrices showed a lower Ca^2+^ peak than organoids grown with a soft matrix, indicating that stiffness regulates ISC differentiation via Ca^2+^ signaling [79]. Other studies have revealed that organoids show different self-renewal and differentiation capacities depending on substrate stiffness and crypt fission, supporting the hypothesis that stiffness influences ISC proliferation and differentiation [77,78].

In addition to the differentiation and proliferation of ISCs, stiffness also affects the intestinal immune system, especially the wound-healing process. Some researchers cultured human colonic fibroblasts on normal or pathologically stiff substrates and found that the density of cells grown on stiff substrates increased and the expression levels of pro-inflammatory genes decreased, which is similar to the wound healing process [80]. Similarly, it was revealed that increased tissue stiffness led to focal adhesion kinase (FAK)-dependent cell proliferation, which is essential for mucosal wound healing [81]. These results suggest that tissue stiffness affects immune responses and tissue repair processes contributing to maintaining intestinal integrity. Moreover, there is some evidence that stiffness is related to immune cells. Macrophages cultured under stiffness conditions have a pro-inflammatory phenotype with lower phagocytosis [92,93]. Bone marrow-derived dendritic cells produce pro-inflammatory cytokines grown on a stiff substrate [94]. Also, stiff dendritic cells affect T-cell activation [95]. Because many immune cells are present in the lamina propria of the intestine, these results suggest that stiffness not only supports tissue repair but also regulates immune cell function, linking mechanical properties to intestinal homeostasis and immune responses (Figure 2).

Some researchers showed the relationship between the gut microbiome and arterial stiffness, highlighting the gut–heart axis. Trimethylamin N-oxide, which is a gut microbiome-derived metabolite, induces aortic stiffness with an increase in intrinsic wall stiffness [96]. However, the specific effects of intestinal stiffness on the gut microbiome remain unclear, so further investigation is needed to establish the relationship.

### 2.3. Compression and Stretch Forces

The rhythmic contractions and expansion of the intestine due to luminal contents or peristaltic waves generate mechanical stress such as compression and stretch. Compression and stretch forces in the intestine and mechanical strain impact ISC behavior. In a study conducted in 2006, small intestinal segments exposed to stretch conditions were longer than those of controls, with a thicker mucosal layer, suggesting that mechanical stretching promotes small intestine proliferation [97]. Furthermore, in a study conducted in 2012, Caco-2 cells cultured with mechanical stretching showed increased cellular heights and altered cytodifferentiation [98]. Moreover, a study using an inflation–collapse model in organoids found that inflation drives a stretch-associated cell state. This process triggers organoid fission, resulting in the loss of Lgr5 expression and the upregulation of Piezo1 [83], suggesting that mechanical stretching can alter the characteristics of ISCs (Table 2). Meng et al. demonstrated that stretching significantly influences ISC behavior. By exposing intestinal organoids to cyclic stretch, they observed that 8% cyclic stretch at a frequency of 0.2 Hz increased organoid size and enhanced stemness. Additionally, organoids grown under cyclic stretching exhibit a higher percentage of Ki67^+^ cells and elevated SOX9 expression, which is a stem cell marker indicating stem cell proliferation. This is accompanied by the activation of the Wnt/β-catenin signaling and the upregulation of the expression of Lgr5 and Olfm4 [84]. Similar to mechanical stretching, other researchers treated intestinal organoids with PEG to apply volumetric compression. They observed that volumetric compression can enhance cytosolic β-catenin accumulation and intracellular crowding. Elevated intracellular crowding leads to higher expression of target genes of the Wnt/β-catenin signaling pathway, such as *LGR5*, *AXIN2*, and *SOX9*, thereby maintaining elevated Wnt/β-catenin signaling. As a result, volumetric compression can promote the self-renewal of ISCs (Table 2) [61].

There is some evidence that compression and stretch can affect the immune system. In 2020 it was revealed that these mechanical forces influence neuron activity, inducing the immediate spike discharge of enteric neurons, which affects their immunoreactivity [99]. To investigate the direct effect of stretch, some researchers applied mechanical deformation and found that it leads to the depolarization of neutrophils, thereby influencing cytokine production [100]. Moreover, bone marrow-derived macrophages recognize cyclic compression through PIEZO1. PIEZO1 mediates Ca^2+^ influx, driving macrophages to express pro-inflammatory characteristics [101]. These findings suggest the critical role of compression and stretch in regulating immune cell function.

Beyond their effects on the immune system, compression and stretch may indirectly influence the gut microbiome. For instance, studies have revealed that when cyclic stretching is interrupted, bacterial overgrowth, such as that found in IBD patients, is observed. In addition, immune cells and LPS together stimulate the release of inflammatory cytokines (Figure 2) [102]. Although direct links between compression or stretch and the microbiome are limited, these results suggest that the gut microbiome is also affected by compression or stretch.

### 2.4. Other Forces

Changes in hydrostatic pressure mediated by the fluid content within the GI tract alter digestive capability and smooth muscle contractile dysfunction. Moreover, hydrostatic pressure plays an important role in cell proliferation and differentiation [103,104,105,106]. Other researchers applied mechanical strain to mouse models by implementing a spring. They found that strain enhanced maturation; therefore, mechanical force-exposed tissues exhibited higher villus height [107]. However, in contrast to other mechanical forces, the influence of hydrostatic pressure and strain on biological processes in the intestine has not yet been established. As the intestine oversees digestion, many mechanical strains in the intestine that influence cell signaling pathways, intestinal homeostasis, and intestinal diseases remain unestablished. Significant efforts have been made to elucidate the impact of mechanical forces on the behavior of intestinal epithelial cells. Gut-on-chip models or organoids are typically used to mimic mechanical forces. Moreover, a computational program that predicts the magnitude of mechanical strength required for lengthening the intestine was developed and applied to patients with short bowel syndrome, considering wall thickness and intestinal radius [108]. These models will help to elucidate the influence of other mechanical or multiplex forces on the intestine.

## 3. Association Between Inflammatory Diseases and the Intestinal Environment

### 3.1. Importance of ECM

Intestinal epithelial cells constantly interact with the ECM, including its components such as integrins, laminin, collagen, fibronectin, and glycosaminoglycans (GAGs) [109]. The remodeling of the ECM surrounding the intestinal crypts is crucial for maintaining intestinal homeostasis. ECM remodeling involves a dynamic series of processes, including the synthesis, deposition, and degradation of ECM components. The key components in this process include matrix metalloproteinases (MMPs), inhibitors of metalloproteinases, and other proteolytic enzymes that regulate ECM turnover [110]. ECM remodeling plays a key role in establishing and maintaining stem cell niches and wound repair [110]. In contrast, abnormal ECM remodeling leads to dysregulated cell proliferation, differentiation, and death, resulting in tissue fibrosis and cancer. For example, the excessive degradation of ECM, such as collagen or laminin, affects epithelial cell adhesion, compromising epithelial integrity and intestinal barrier function, resulting in increased intestinal permeability and intestinal inflammatory diseases [111]. The degradation of ECM by the gut microbiome accelerates inflammation in a dextran sodium sulfate (DSS)-induced colitis model [112]. In addition, an increase in the levels of MMP-9 mediates the degradation of ECM components, causes damage to the intestinal epithelial barrier, induces inflammation, and is related to the onset of inflammatory diseases [113].

However, insufficient ECM degradation or excessive ECM deposition leads to ECM accumulation and fibrosis, which can alter the mechanical properties of the intestinal microenvironment, resulting in intestinal diseases [113,114]. This process disrupts normal cellular signaling, inhibits the regeneration of intestinal epithelial cells, and leads to ECM stiffness [87,115]. The dual nature of ECM remodeling, which is essential for normal gut function but detrimental when dysregulated, highlights the importance of understanding the precise molecular mechanisms that control this dynamic system.

### 3.2. Remodeling of ECM in IBD

Chronic inflammatory diseases such as IBD significantly alter the structural and functional integrity of the intestinal epithelial barrier [116]. Constant inflammation leads to fibrosis and disrupts the function and composition of the intestinal epithelial cells. In addition, when the intestinal epithelial barrier loses its function, microbial infiltration is initiated, leading to immune cell activation, the exacerbation of inflammation, and local ECM remodeling [117]. These interactions between inflammation, ECM, and immune cells are observed in intestinal inflammatory diseases and emphasize the complex interplay between mechanical properties and tissue pathology.

Accumulating evidence indicates that inflammation in the intestine leads to uncontrolled ECM remodeling and alters the mechanical properties. Yui et al. demonstrated that DSS-induced colitis leads to significant ECM remodeling in a YAP/TAZ signaling-dependent manner [118]. ECM remodeling is a key feature of IBD and is associated with disease progression and inflammation. In particular, as IBD progresses, proteolytic activity increases, leading to the breakdown of ECM components and tissue damage [119,120]. The key players in ECM degradation include the family of MMPs that break down ECM components. MMP activity increased in both patients with IBD and DSS-treated mice, resulting in the accumulation of collagen degradation products, including proline-glycine-proline (PGP). Furthermore, MMP contributes to intestinal damage by amplifying inflammation [121]. Additionally, MMP-9 levels are increased in patients with IBD and are associated with elevated intestinal permeability [122]. Moreover, increased MMP activity contributes to inflammatory processes that affect the adhesion and migration of immune cells [123,124]. In addition to ECM degradation, ECM deposition is associated with IBD. Chronic inflammation induces fibrosis, which causes stiffness, and is a consequence of IBD. Liu et al. revealed that mast cell infiltration is increased in DSS-induced fibrosis models. Furthermore, mast cells are involved in ECM deposition by releasing tryptase. Tryptase activates the PAR-2/Akt/mTOR pathway and triggers fibroblast differentiation, promoting intestinal fibrosis and damage [125]. Moreover, tissue stiffness caused by inflammation influences the fate of ISCs [76]. These findings demonstrate the importance of the interplay between ECM degradation and deposition in IBD, which alters the mechanical properties of the intestinal environment. Excessive protease activation disrupts the intestinal barrier function; however, ECM deposition induces fibrosis, leading to stiffness and the modulation of ISC function, highlighting the importance of targeting ECM remodeling for IBD treatment.

### 3.3. Mechanical Forces in Inflammation in the Intestine

Mechanical forces, including stretching, compression, and shear stress, are also associated with inflammation. For example, mechanical stretching increases the levels of pro-inflammatory mediators such as inducible nitric oxide synthase, IL-6, and monocyte chemoattractant protein-1 (MCP-1), amplifying local inflammatory responses, recruiting immune cells, and exacerbating inflammation [126]. These findings suggested that mechanical forces play an important role in inflammation. Some researchers have revealed that patients with IBD experience different mechanical forces than healthy individuals. Roifman et al. revealed that patients with IBD showed lower shear stress-induced reactive hyperemia than a healthy group, indicating greater cardiovascular risk and endothelial dysfunction [127]. Other researchers have suggested that disturbances in autophagy cause IBD, and shear stress can induce non-canonical autophagy [63]. Furthermore, Geesala et al. found that treatment with exclusive enteral nutrition, a liquid diet that can reduce mechanical stress in the intestine by reducing lumen distension, inhibited IL-6 production and Th17 differentiation in a rat model with colitis [128,129].

The relationship between mechanical forces and inflammation is not limited to IBD but also extends to CRC. A study conducted in 2015 revealed that the compression generated by the expansion of hyperproliferative cells, such as CRC cells, affects the surrounding normal epithelial cells. This mechanical force leads to the overexpression of the notch signaling, increasing β-catenin levels. Furthermore, they implanted a magnet in a mouse colon and found an activated Wnt/β-catenin pathway with crypt enlargement and the formation of early tumors [130]. Other researchers found that shear stress disrupted cell-cycle progression in colon cancer cells and regulated the expression of β-catenin [131]. Shear stress is also involved in reducing the migration and invasion of the CRC cell line, SW620, by regulating the cell cycle [132]. These findings emphasize the importance of the mechanical properties of inflammatory diseases in the intestine.

In addition to inflammatory diseases, mechanical forces are associated with IBS. A comparative study revealed that patients with IBS exhibited distinct colonic motility; repetitive distention of the colon induces increased colon motility in patients with IBS in contrast to healthy subjects [133]. These results indicated that mechanical forces are involved in the development of various intestinal disorders.

### 3.4. ECM Remodeling in CRC

ECM remodeling also plays an important role in the tumor microenvironment. In the normal state, the ECM is highly regulated and organized; however, in CRC, the ECM is reorganized and dysregulated [134]. It is also closely associated with cell proliferation and invasion. ECM remodeling around the tumor microenvironment leads to stiffness, creating a suitable environment for CRC invasion and vascularization. Colon stiffness may serve as a novel predictive marker for invasion [135]. A cohort analysis of 18 patients revealed that tumor areas were stiffer and the mutations or its stage of CRC were related to increased tissue stiffness [136]. Furthermore, the ECM of CRC shows significantly higher stiffness than that of control tissues, with increased levels of GAGs and collagen fibers, suggesting an important role for GAGs in ECM remodeling [134]. Additionally, in CRC tissues and cell lines, extracellular matrix protein 1 (ECM1) is upregulated and positively related to tumor size and the TNM stage. The inhibition of ECM1 disrupts the growth, invasion, and migration of CRC, and the overexpression of ECM1 enhances CRC progression, suggesting that the ECM increases CRC invasion [137]. However, other researchers have suggested that CRC is correlated with dysregulated ECM remodeling and increased stiffness but is negatively related to CRC migration and invasion. For example, collagen is upregulated in the tissues of CRC patients, and increased ECM density inhibits the migration and invasion of CRC cell lines [138]. Zhu et al. also reported that CRC tissues and serum show higher levels of fibulin-1, an ECM component related to lymph node invasion. The stage of CRC in patients with elevated expression of fibulin-1 is I or II, but patients with lower levels of fibulin-1 show lymph node involvement and metastasis [139]. These results indicated that tissue stiffness disrupts CRC cell invasion and metastasis.

**Figure 3:**
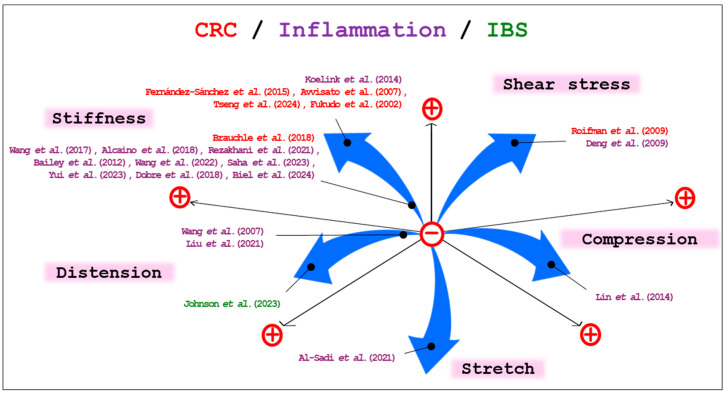
Mechanical forces and intestinal disease. This schematic shows the connections between mechanical stress and intestinal diseases. “−” means a negative correlation, and “+” means a positive correlation between stress and intestinal diseases [85,86,109,115,116,117,118,119,120,121,122,123,124,125,126,127,129,130,131,132,133,134].

Although it is unclear how tissue stiffness affects CRC metastasis, these findings suggest that the mechanical properties of the intestine are significantly associated with intestinal diseases, and mechanical feedback loops play a key role in chronic inflammatory conditions, such as IBD or CRC (Table 3 and Figure 3). By understanding how tissue stiffness, compression, and shear stress affect cellular behavior and ECM remodeling, we can obtain insights into the mechanisms underlying disease progression and potential therapeutic strategies.

## 4. Conclusions and Perspectives

The intestine is constantly exposed to various mechanical forces that influence the behavior and homeostasis of intestinal epithelial cells, including shear stress, stiffness, compression, and stretching. These forces play crucial roles in maintaining intestinal integrity by regulating the proliferation and differentiation of ISCs via mechanotransduction signaling pathways. In addition, dysregulated mechanical forces are associated with the progression of intestinal diseases, including IBS, IBD, and CRC. Over the past few decades, we have attempted to develop and mimic the actual human intestine via in vitro and ex vivo models using advanced techniques, such as gut-on-chip models, 3D organoids, and computational programs. However, the development of experimental tools that model the mechanical forces of the intestinal environment is still limited by various constraints. Therefore, a convergent mechanobiology study that considers the immune system, gut microbiome, and mechanical stress together can expand our understanding of the intestinal microenvironment and aid in the development of novel therapeutic strategies for intestinal diseases.

## Figures and Tables

**Table 3:** The correlation between mechanical forces and intestinal diseases.

Mechanical Forces	Intestinal Diseases	
	Positive	Negative
Shear stress	-	[123,127]
Stiffness	[121,130,131,132,133]	[85,86,109,115,116,117,118,119,120,134]
Compression	[126]	-
Stretch	[122]	-
Distension	[129]	[124,125]

## Abbreviations

The following abbreviations are used in this manuscript:

IECs	intestinal epithelial cells
IBS	irritable bowel syndrome
IBD	inflammatory bowel disease
YAP	Yes-associated protein
ECM	extracellular matrix
IL	interleukin
CCK	cholecystokinin
IEC	intestinal epithelial cell
ZO	zonula occludens
BMP	bone morphogenic protein
TAZ	transcriptional co-activator with PDZ-binding motif
ISC	intestinal stem cell
AMP	antimicrobial peptide
TA	transit-amplifying zone
GAG	glycosaminoglycan
MMP	matrix metalloproteinase
CRC	colorectal cancer
ECM1	extracellular matrix protein 1
DSS	dextran sodium sulfate
MCP-1	monocyte chemoattractant protein-1
